# Vertebral Vascular Canal Dysplasia in Three Brachycephalic Breeds and Its Relationship With BOAS Grade

**DOI:** 10.1111/vru.70236

**Published:** 2026-09-18

**Authors:** Margarida de Almeida Coelho, Thomas Maddox, Jane F. Ladlow, Marie‐Aude Genain

**Affiliations:** ^1^ Department of Veterinary Medicine University of Cambridge Cambridge UK; ^2^ Small Animal Teaching Hospital University of Liverpool, Neston Wirral UK; ^3^ Granta Veterinary Specialists Cambridge UK

**Keywords:** basivertebral canal, canine, vertebral anomaly, vertebral venous system

## Abstract

The relationship between vertebral vascular canal dilation and raised intra‐abdominal and intrathoracic pressures has been observed in humans. Vertebral vascular canal dysplasia (VVCD) has recently been reported as a vertebral anomaly in brachycephalic dogs. This retrospective, single‐institutional study aimed to estimate the prevalence of VVCD number and type in thoracic vertebrae of three brachycephalic breeds and a non‐brachycephalic control group, and to correlate the findings with the brachycephalic obstructive airway syndrome (BOAS) functional grading. Thoracic CT studies of French Bulldogs, English Bulldogs, and Pugs that had previously undergone BOAS assessment were retrospectively reviewed. Non‐brachycephalic dogs that underwent a thoracic CT scan were matched by weight with the BOAS group and randomly selected to be part of the control group. The prevalence of VVCD involving ≥1 thoracic vertebra was 20% (6/30) in the control group, 56.9% (136/239) in French Bulldogs, 95.3% (41/43) in English Bulldogs, and 87.3% (55/63) in Pugs. French Bulldogs had significantly fewer vertebrae affected than the other brachycephalic breeds (*p *< 0.001). BOAS grade III dogs had more affected vertebrae; however, this was not statistically significant on multivariable analysis. The difference between the VVCD type and BOAS grades was not significant (*p* = 0.33). As BOAS severity was not correlated with either the presence or type of VVCD, no evidence was found to support a causative link between BOAS grading and the degree of vertebral vascular canal dilation. The potential role of raised intrathoracic or intra‐abdominal pressures in the development of VVCD therefore remains hypothetical.

**Abbreviations**
BOAS: brachycephalic obstructive airway syndrome
ETT: exercise tolerance test
QVSH: Queen's Veterinary School Hospital
VVCD: vertebral vascular canal dysplasia
VVS: vertebral venous system

## 1 Introduction

Brachycephalic obstructive airway syndrome (BOAS) is a conformation‐related disorder that affects extremely brachycephalic dogs such as Pugs, French Bulldogs, and English Bulldogs. It is primarily characterized by respiratory problems involving partial or complete obstruction of the upper airways [1, 2, 3]. The shortening of the skull in these dogs leads to compression of the nasal and pharyngeal air passages; however, the changes in the soft tissues are not proportional, resulting in a relative excess of soft tissue blocking the airflow. Lesion sites are breed‐specific, with Pugs and French Bulldogs being more frequently affected by stenotic nares and rostral aberrant turbinates, whereas in English Bulldogs tracheal hypoplasia is the predominant lesion [4]. These anatomical malformations cause airway resistance and increased negative pressure within the upper airways during respiration [1]. Increased transdiaphragmatic pressure gradient and intra‐abdominal pressures have also been described in brachycephalic dogs with BOAS suffering from concomitant gastrointestinal diseases; however, this has not been explored in dogs without gastrointestinal disturbances [5, 6]. A functional grading system based on clinical evaluation of animals before and after a 3‐min exercise tolerance test (ETT) has been developed to evaluate BOAS severity (Table 1). Four grades integrate the BOAS functional grading system—grade 0 dogs are unaffected and therefore BOAS free, grade I dogs are mildly affected, showing mild respiratory noise but unaffected exercise tolerance, grade II dogs have moderate BOAS and require medical attention such as weight control, surgical intervention, or both, and grade III dogs have severe BOAS and require immediate surgical intervention [7].

**TABLE 1:** Brachycephalic obstructive airway syndrome (BOAS) functional grading system based on respiratory signs before and after an exercise tolerance test (ETT).

	Respiratory noise	Inspiratory effort	Dyspnea/Cyanosis/Syncope
Grade 0			
Pre‐ETT	Not audible	Not present	Not present
Post‐ETT	Not audible	Not present	Not present
Grade I			
Pre‐ETT	Not audible to mild	Not present	Not present
Post‐ETT	Mild	Not present to mild	Not present
Grade II			
Pre‐ETT	Mild to moderate	Mild to moderate	Not present
Post‐ETT	Moderate to severe	Moderate to severe	Mild dyspnea; cyanosis or syncope not present
Grade III			
Pre‐ETT	Moderate to severe	Moderate to severe	Moderate to severe dyspnea; may or may not present cyanosis. Inability to exercise
Post‐ETT	Severe	Severe	Severe dyspnea; may or may not present cyanosis or syncope

*Source*: Adapted from [7].

Congenital vertebral abnormalities in these breeds are common and frequently described on diagnostic imaging of the vertebral column [8, 9, 10, 11]. Conversely, the presence of vertebral vascular canal dysplasia (VVCD) in the thoracic vertebrae of French and English Bulldogs was only recently characterized in the literature [12]. This anomaly has not yet been described in Pugs. Possible causes for the presence of VVCD in dogs have been proposed. Although the embryological development of these vascular canals remains unclear, it has been suggested that they may form passively following the development of the basivertebral veins. Subsequent distension of these veins could stimulate osteoclastic activity, leading to widening of the canals [12]. Maternal hypoxia has also been suggested as a potential cause for VVCD formation, as it has been previously associated with vertebral abnormalities in other mammalian species [12, 13, 14, 15]. Additionally, both genetic and environmental factors have been hypothesized as playing a role in the development of VVCD [12]. The clinical significance of this vertebral anomaly is also currently unknown.

**FIGURE 1:**
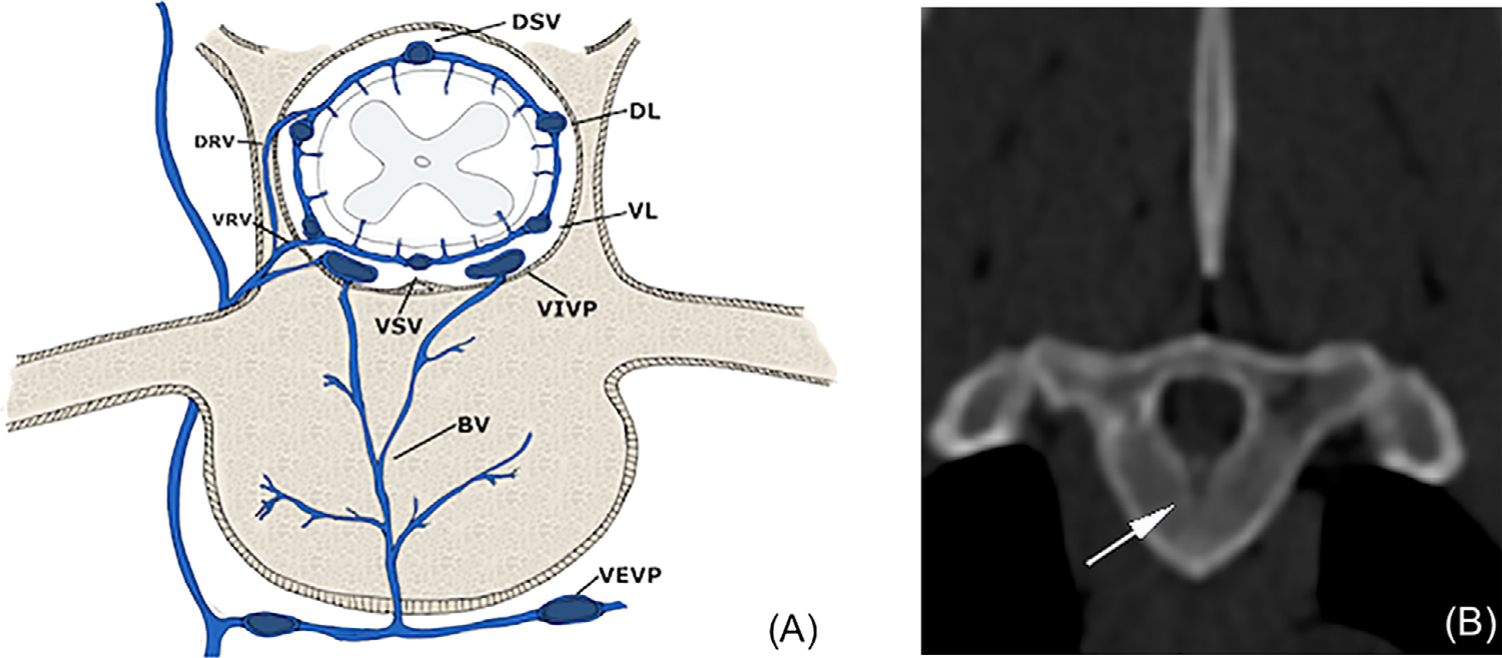
(A) Schematic representation of the canine vertebral venous system anatomy in a transverse section. (B) Transverse CT image of a canine thoracic vertebral body displayed in the bone window. The “Y”‐shaped basivertebral venous canal is indicated by the arrow. BV, basivertebral vein; DL, dorsolateral vein; DRV, dorsal radicular vein; DSV, dorsal spinal vein; VEVP, ventral external vertebral venous plexus; VIVP, ventral internal vertebral venous plexus; VL, ventrolateral vein; VRV, ventral radicular vein; VSV, ventral spinal vein.

The vertebral venous system (VVS) is a valveless system that constitutes an alternative route for venous flow return from the body to the heart. It consists of the internal venous vertebral plexus that lies within the vertebral canal and surrounds the spinal cord, the external venous vertebral plexus, which surrounds the vertebral column, and the basivertebral veins that run horizontally and lie within the vertebrae (Figure 1). The basivertebral veins can be simple or double and arise within the vertebral bodies or from the soft tissues ventral to the vertebrae, projecting dorsally through osseous canals in the vertebral bodies [16]. At the level of the thoracic spine, the basivertebral veins are most commonly absent or single [16]. The flow and direction of blood flow through this valveless system depend on pressure gradients between the intra‐abdominal pressure, intrathoracic pressure, and the pressure in the vertebral canal. Hydrostatic factors and gravitational forces can also affect it [17]. In humans, different shapes and topographic variations of the vertebral vascular canal have been described, with a mean depth of the normal canal corresponding to 12%–27% of the vertebral body height, increasing caudally [18, 19]. In dogs, the CT appearance of the normal vertebral vascular canal has been poorly described. It is normally seen as a Y‐shaped lucency at the mid‐portion of the lumbar vertebral body, and, in other locations, it has anecdotally been described as a very thin bony canal [12, 20]. In human patients, a potential relationship between dilation of various portions of the VVS and raised intrathoracic and intra‐abdominal pressures has been described [17, 21, 22, 23, 24]. In these studies, patients presented with different conditions such as venous obstruction, portal hypertension, and raised intracranial pressure. The increase in size of the vascular vertebral canal has also been suggested as a result of chronic venous dilation [22].

The authors hypothesized that dogs with a higher BOAS functional grading, and therefore with chronic increase in thoracic airway pressure and potentially increased intra‐abdominal pressures, would have a higher prevalence of VVCD and/or more severe dilations. This study aimed to estimate the prevalence of VVCD number and type in thoracic vertebrae of three brachycephalic breeds and a non‐brachycephalic control group and to correlate the findings with the BOAS functional grading.

## 2 Materials and Methods

### 2.1 Selection and Description of Subjects

The present study was a single‐center, retrospective study. Ethical approval was obtained from the Cambridge Univeristy Ethics and Welfare Commitee (reference CR669).

The database of medical records of canine patients seen at the Queen's Veterinary School Hospital (QVSH) for BOAS assessment between January 2014 and September 2022 was screened. Criteria for case inclusion were (1) having a clinical diagnosis of BOAS and known functional grading; (2) being a French Bulldog, English Bulldog, or Pug; and (3) having undergone a thoracic CT study. Cases were excluded if they had a thoracic CT study performed at another institution prior to referral. For the control group, the QVSH database was searched for mesocephalic dogs that underwent a thoracic CT study during the same period, regardless of the primary complaint, and were weight‐matched to the brachycephalic breeds, being excluded if no weight was available on the clinical records. Thirty cases were then selected randomly using an online random number generator to integrate the control group.

### 2.2 Data Recording and Analysis

Patients’ records were reviewed, and breed, age, gender, neutered status, weight, body condition score, functional BOAS grading, and, when available, CT imaging follow‐up were recorded. Concurrent conditions were not recorded.

The CT images were retrieved and retrospectively reviewed by a junior clinician (M.C.) working under the supervision of an ECVDI‐certified veterinary radiologist (M‐A.G.), using an open‐source 64‐bit medical DICOM image viewer (Horos version 3.3.6, Purview, Geneva, Switzerland; iMac Retina 4K, Apple Inc., California, USA). At the time of image review, the two assessors were blinded to patient data and previous imaging reports. Multiplanar reconstruction was performed to generate transverse, sagittal, and dorsal plane reconstructions. The images were displayed using a bone window (window level 500 and width 2500 HU). The CT images were assessed for the presence, number, and location of abnormal vertebrae, as well as for VVCD number, location, and characteristics. When a follow‐up CT was available, VVCD number, location, and characteristics were also recorded. Anomalous or malformed vertebrae were not excluded. The VVCD were classified using the method described by Santifort et al. in [25]. When present, they were evaluated for whether the defect involved a maximum of 50% (Score “A”), 50%–100% (Score “B”), or 100% (Score “C”) of the dorsoventral height of the vertebral body and whether the anomaly was single (midline or unilateral; Subscore “s”), double (bilateral; Subscore “d”), or complex (double and converging to single or deviating shape; Subscore “c”) [12]. All measurements were performed on transverse images using the linear measuring function of the image viewer. In cases where VVCD was found in abnormally shaped vertebrae, the dorsoventral height of the vertebral body was measured at its greatest point.

To evaluate interobserver agreement, a subset of 30 randomly selected dogs, using the same online random number generator, was reviewed by a second reviewer, and the same details were recorded.

### 2.3 Statistics

Statistical analyses were performed by a PhD‐trained statistician in epidemiology (T.M) using commercially available software (IBM SPSS Statistics 30 and MLwiN version 3.10.0). Continuous variables (age, weight, and VVCD number) were assessed for distribution using graphical analysis and with the Kolmogorov–Smirnov test of normality. For each brachycephalic breed and control group, the median and range were calculated for age and weight. Prevalence of VVCD number and type was calculated for each brachycephalic breed and the control group. The number of VVCD per dog was not normally distributed. To evaluate if there was an association between this and any of the recorded independent variables (age, sex, weight, breed, control or case status, and BOAS grade), a Poisson regression was performed. As dogs could have multiple VVCD of different types, VVCD types were clustered within dogs, and to account for this, a multilevel multinomial logistic regression analysis was performed to assess for association between the type of VVCD and the same independent variables, with dog included as a random intercept term. For both regression analyses, any variables indicating a possible association (*p *< 0.2) with the dependent variable on univariable analysis were considered for inclusion in a multivariable model. For the evaluation of significance, the *p*‐value was set at <0.05. To assess interobserver agreement for the number of VVCD identified per dog, weighted kappa analysis (using linear weighting) was calculated. Cohen's kappa was used to assess interobserver agreement for the vertebra specifically affected with VVCD and the type of VVCD. The 95% confidence intervals (95%CI) were calculated for all kappa values, and kappa was interpreted according to previously suggested guidelines [26].

## 3 Results

### 3.1 Study Population

Over the study period, 353 dogs met the inclusion criteria for the brachycephalic group. Eight cases were excluded; seven cases underwent a CT study at another institution, and one case declined to be involved in clinical research. A total of 345 dogs were then included in the study, of which 239 (69.3%) were French Bulldogs, 63 (18.3%) were Pugs, and 43 (12.5%) were English Bulldogs. Patient demographics are presented in Table 2.

**TABLE 2:** Patient's signalment and brachycephalic obstructive airway syndrome (BOAS) grading per breed.

	French Bulldog	Pug	English Bulldog
Age			
Mean	2 years	2 years 7 months	2 years 1 month
Range	6 months–10 years	7 months–10 years	9 months–5 years 3 months
Weight (kg)			
Mean	12.2	8	24.3
Range	5.8–19.5	5.1–14.85	14.6–34.5
Sex			
Entire females	22	19	5
Neutered females	35	22	6
Entire males	116	11	20
Neutered males	66	12	11
BOAS grading			
I	19	9	2
II	141	28	21
III	79	26	20

All brachycephalic dogs underwent BOAS assessment and were graded from 0 to III by different trained observers. There were no grade 0 animals. Thirty dogs (8.7%) had BOAS grade I, of which 19 were French Bulldogs, 9 were Pugs, and 2 were English Bulldogs. There were 190 dogs (55.1%) graded BOAS grade II, with 141 being French Bulldogs, 28 Pugs, and 21 English Bulldogs. The remaining 125 dogs (36.2%) were BOAS grade III, of which 79 were French Bulldogs, 26 were Pugs, and 20 were English Bulldogs (Table 2).

During the same period, 77 dogs met the criteria to be included in the control group, of which 15 were excluded due to lack of body weight for review. Out of the 30 randomly selected dogs, there were 22 Cocker Spaniels (73.3%), three West Highland White Terriers (10.0%), one Beagle (3.3%), one Border Terrier (3.3%), one Cockerpoo (3.3%), one Miniature Schnauzer (3.3%), and one Scottish Terrier (3.3%). The patient's age ranged from 11 months to 13 years old (median: 8 years old). The median weight was 14 kg (range 7.3–22.5). There was one entire female, 12 neutered females, six entire males, and 11 neutered males.

All CT images were acquired using the same system (Toshiba Aquilion 16‐slice helical scanner), with slice thickness 1.0 mm, 100 kV, variable mA, and rotation time 0.5 s. Contrast medium was administered in some patients, but all measurements were performed on pre‐contrast images. All patients were under general anesthesia and in sternal recumbency.

### 3.2 VVCD Number

**FIGURE 2:**
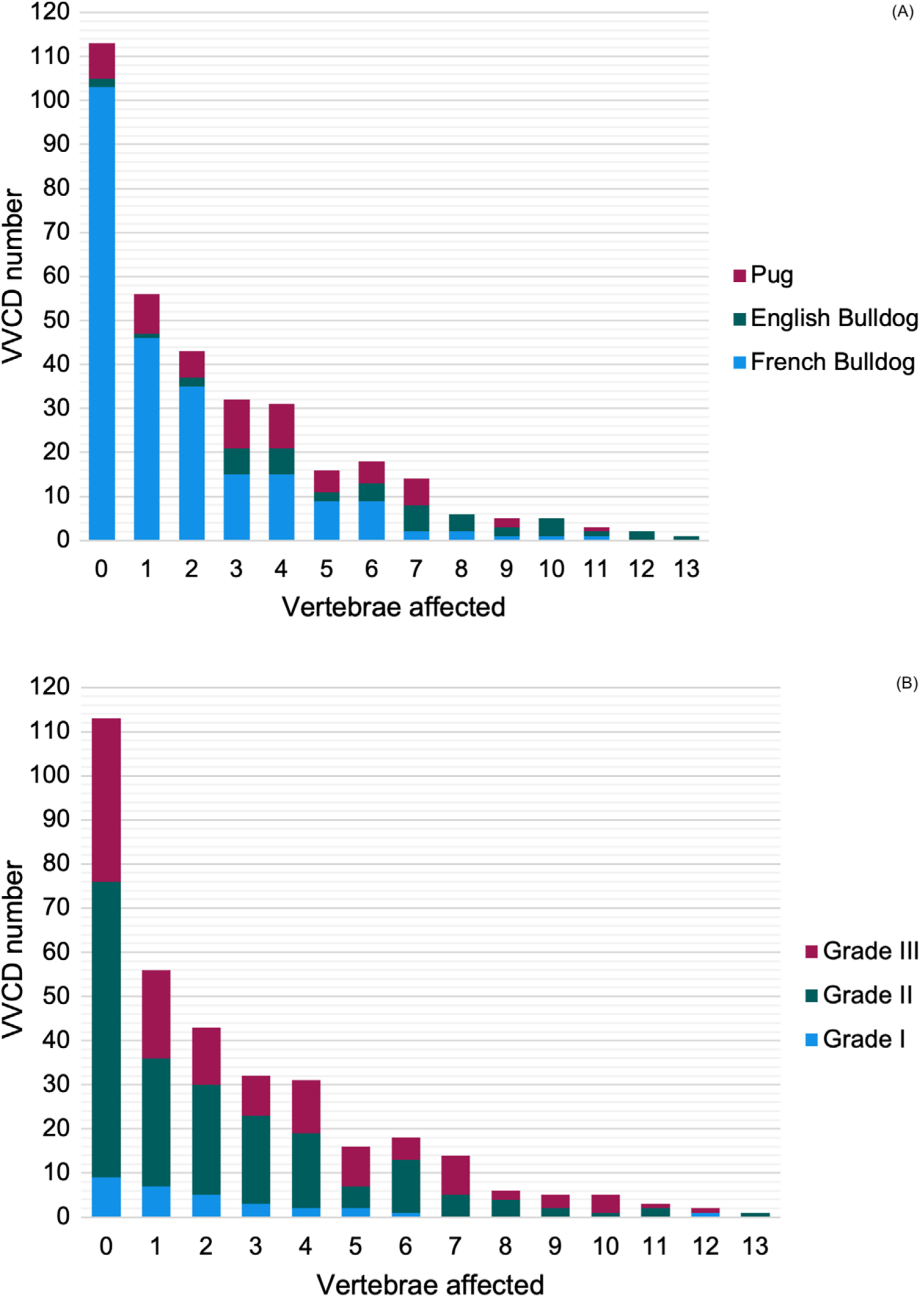
Number of vertebrae affected with VVCD per breed (A) and BOAS grade (B). BOAS, brachycephalic obstructive airway syndrome; VVCD, vertebral vascular canal dysplasia.

The median number of vertebrae affected with VVCD was two (range 0–13). In 113/345 cases, no VVCD was detected (33%), and in only one case all vertebrae were affected (Figure 2). The patient who had all vertebrae affected was an English Bulldog with BOAS grade II. There was no statistical significance between the number of vertebrae affected with VVCD and the patient's age (*p *= 0.22) or sex (*p *= 0.34). There was a significant difference between weight and number of affected vertebrae, with dogs with lower body weight having fewer vertebrae affected (*p *< 0.001).

**FIGURE 3:**
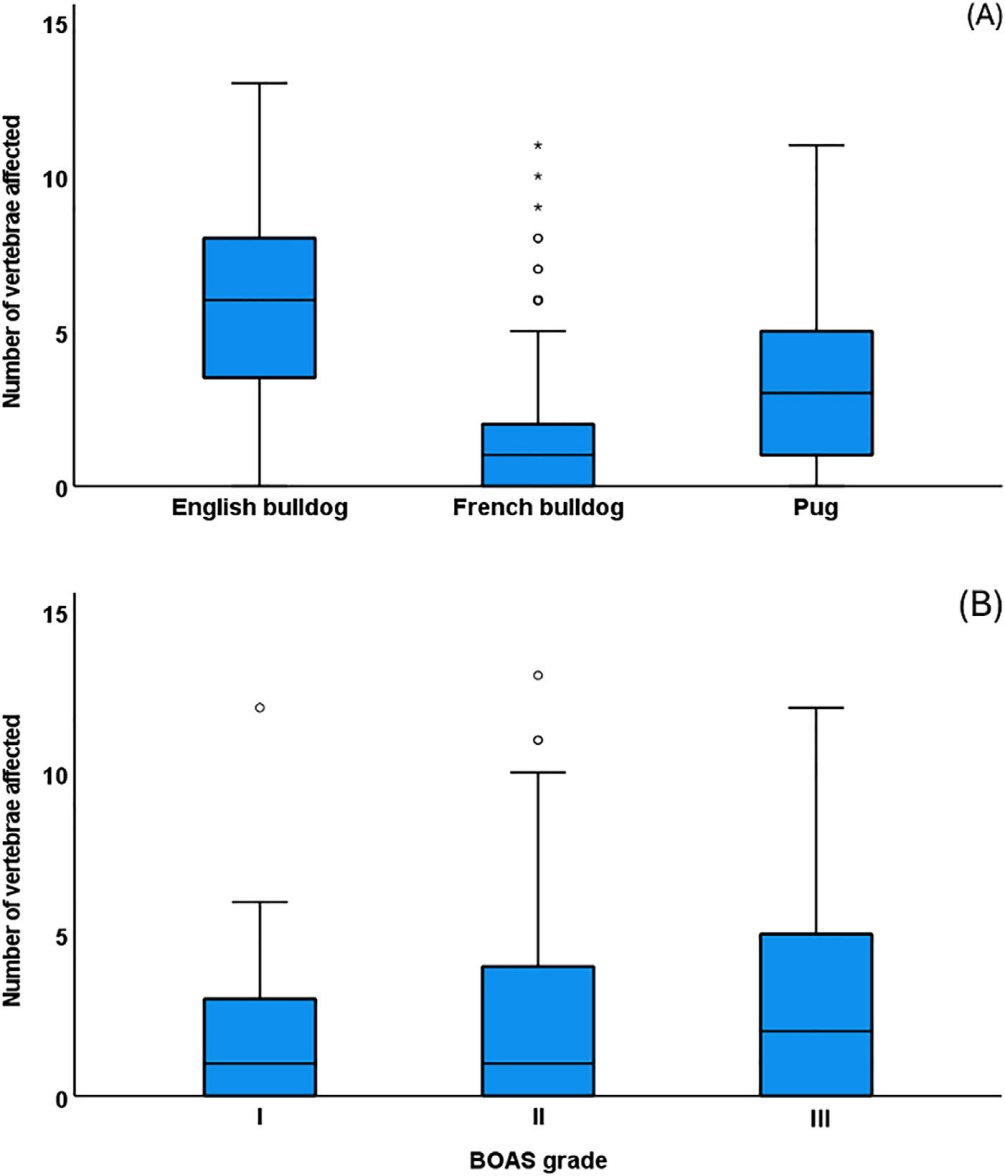
Comparison of the number of vertebrae affected with VVCD per breed (A) and BOAS grade (B). BOAS, brachycephalic obstructive airway syndrome; VVCD, vertebral vascular canal dysplasia.

The median number of vertebrae affected was one (range 0–11) in French Bulldogs, three in Pugs (range 0–11), and six in English Bulldogs (0–13). The difference in vertebrae affected was statistically significant (*p *< 0.001), with English Bulldogs having more than both other breed groups (*p *< 0.001) (Figure 3).

For animals with BOAS grades I and II, the median number of vertebrae affected with VVCD was one (range 0–12 and 0–13, respectively); for animals with BOAS grade III, it was two (range 0–12), with the latter having significantly more vertebrae affected (*p *= 0.001) (Figure 3).

In the control group, the median number of vertebrae affected was zero (range 0–2). This was statistically different from the brachycephalic group (*p *< 0.001).

Weight, breed, and BOAS grade were included in the multivariable analysis, but when controlling for weight and BOAS grade, only breed showed a significant association (*p* < 0.001) with the number of VVCD‐affected vertebrae.

### 3.3 VVCD Type

**FIGURE 4:**
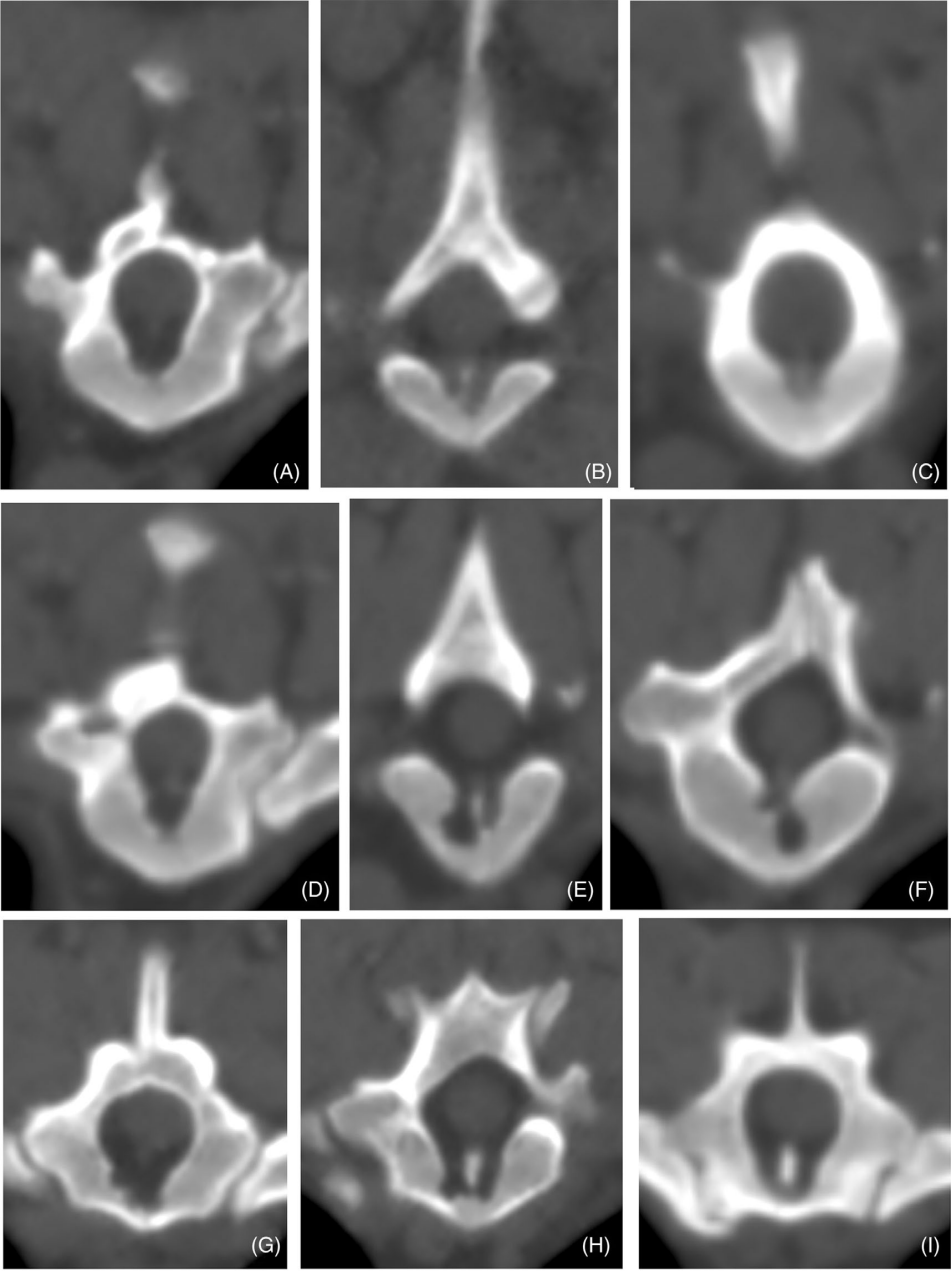
Transverse CT images of canine thoracic vertebrae affected by VVCD involving a maximum of 50% of the dorsoventral vertebral body height (Score “A”; A–C), between 50% and 100% (Score “B”; D–F), and 100% (Score “C”; G–I). The anomalies were also classified as being single (Subscore “s”; A, D, G), double (Subscore “d”; B, E, H), or complex (Subscore “c”; C, F, I). The images are displayed using a bone window (window level 500 and width 2500 HU). (A) Score “As”; (B) Score “Ad”; (C) Score “Ac”; (D) Score “Bs”; (E) Score “Bd”; (F) Score “Bc”; (G) Score “Cs”; (H) Score “Cd”; (I) Score “Cc”. VVCD, vertebral vascular canal dysplasia.

In total, 831 vertebrae were affected with VVCD throughout the brachycephalic group, of which 515 scored “A” (62.0%), 271 scored “B” (32.6%), and 45 scored “C” (5.4%) (Figure 4).

There was no statistical difference between the type of VVCD and the patient's age (*p* = 0.58) or sex (*p* = 0.80), but there was for weight (*p *< 0.001), with increasing weight associated with types B and C versus A.

**FIGURE 5:**
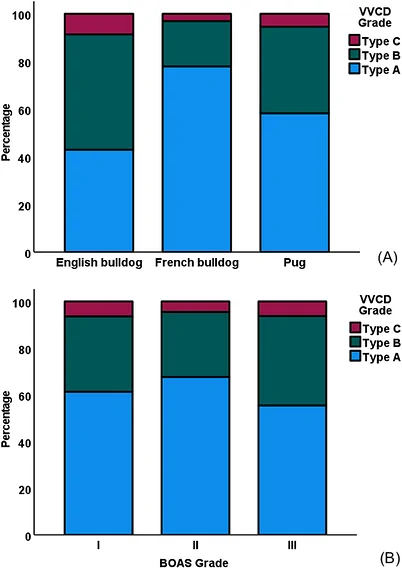
Comparison of type of VVCD per breed (A) and BOAS grade (B). BOAS, brachycephalic obstructive airway syndrome; VVCD, vertebral vascular canal dysplasia.

French Bulldogs had 357/831 vertebrae affected (43.0%), of which 278 scored “A” (77.9%), 68 scored “B” (19.0%), and 11 scored “C” (3.1%). English Bulldogs had 254/831 vertebrae affected (30.1%), of which 109 scored “A” (42.9%), 123 scored “B” (48.4%), and 22 scored “C” (8.7%). Pugs had 220/831 vertebrae affected (26.5%), of which 128 scored “A” (58.2%), 80 scored “B” (36.4%), and 12 scored “C” (5.4%) (Table 3; Figure 5). The difference between breeds was statistically significant (*p *< 0.001).

**TABLE 3:** Type of vertebral vascular canal dysplasia (VVCD) per breed.

VVCD type	French Bulldog	Pug	English Bulldog
A	278/357 (77.9%)	128/220 (58.2%)	109/254 (42.9%)
B	68/357 (19.0%)	80/220 (36.4%)	123/254 (48.4%)
C	11/357 (3.1%)	12/220 (5.4%)	22/254 (8.7%)

*Note*: These proportions represent breed‐level distributions of VVCD type and not population‐level prevalences.

Of the 831 total affected vertebrae, 70 vertebrae were affected in animals with BOAS grade I (8.4%), 417 vertebrae were affected in animals with BOAS grade II (50.2%), and 352 vertebrae were affected in animals with BOAS grade III (42.4%). In BOAS grade I patients, 38/70 of the VVCD scored “A” (54.3%), 20/70 scored “B” (28.6%), and 4/70 scored “C” (5.7%). In BOAS grade II patients, 282/417 of the VVCD scored “A” (67.6%), 116/417 scored “B” (27.8%), and 19/417 scored “C” (4.6%). In BOAS grade III patients, 195/352 of the VVCD scored “A” (55.4%), 135/352 scored “B” (38.4%), and 22/352 scored “C” (6.2%) (Table 4, Figure 5). The difference between the type of VVCD and BOAS grades was not significant (*p* = 0.33).

**TABLE 4:** Type of vertebral vascular canal dysplasia (VVCD) per brachycephalic obstructive airway syndrome (BOAS) grade.

VVCD type	BOAS grade I	BOAS grade II	BOAS grade III
A	38/70 (54.3%)	282/417 (67.6%)	195/352 (55.4%)
B	20/70 (28.6%)	116/417 (27.8%)	135/352 (38.4%)
C	4/70 (5.7%)	19/417 (4.6%)	22/352 (6.2%)

*Note*: These proportions represent grade‐level distributions of VVCD type and not population‐level prevalences.

Weight and breed were included in the final multivariable model, but, when controlling for weight, only breed was significant (*p *= 0.031), with French Bulldogs having more type “A” VVCD and fewer type “B” than Pugs and English Bulldogs, and fewer type “C” than English Bulldogs.

In the control group, all affected vertebrae had a VVCD score of “A” (7/7).

There was moderate agreement between observers for the number of VVCD per dog (weighted kappa = 0.49; 95%CI 0.29–0.69) and fair agreement between observers for both vertebrae affected with VVCD (kappa = 0.37; 95%CI 0.26–0.49) and type of VVCD identified (kappa = 0.30; 95%CI 0.20–0.41).

### 3.4 CT Follow‐Up

During the study period, 11/345 (3.2%) dogs had follow‐up CT studies at different time points following surgical intervention to address their BOAS. Time between both studies ranged from 35 to 1362 days. All dogs had reduced BOAS grading at the time of the follow‐up CT study compared with initial imaging. Four dogs initially classified as having BOAS grade II were then reported to be BOAS grade 0 (1/4) or grade I (3/4). The remaining dogs (7/11) were initially classified as having BOAS grade III, reducing to BOAS grade I (1/7) or BOAS grade II (6/7) at the time of follow‐up CT. No changes were seen in either VVCD number or type when compared to preoperative CT studies.

## 4 Discussion

The present study reiterates the presence of VVCD as a vertebral malformation in brachycephalic dogs and correlates these findings with the patient's BOAS grading.

Although the presence of VVCD has only been reported in the veterinary literature as a vertebral anomaly found in brachycephalic breeds, no previous comparison between the prevalence of VVCD in brachycephalic and non‐brachycephalic breeds has been performed [12]. In this study, the prevalence of VVCD was found to be significantly higher in brachycephalic breeds compared to the control group, supporting the belief that this anomaly is associated with brachycephalic dog breeds. Dogs with BOAS grade III had a significantly greater number of vertebrae affected by VVCD compared with dogs in the lower BOAS grades. However, this association was not maintained in the multivariable analysis. After controlling for BOAS grade, breed remained the only significant factor, with English Bulldogs exhibiting a higher number of affected vertebrae than the other breeds. No significant differences were identified between the type of VVCD and the patients’ BOAS grading. These findings suggest that neither the number nor severity of VVCD reflects the extent of physiological compromise due to BOAS. It is also important to note that 62% of the affected vertebrae in brachycephalic dogs were assigned a VVCD score of “A,” indicating that more than half exhibited only mild vertebral vascular canal dilation. In human medicine, the literature suggests a possible association between raised intra‐abdominal and intrathoracic pressures with VVS dilation in patients with different chronic comorbidities not analogous to BOAS in dogs [17, 21, 22, 23, 24]. The results of this study did not reveal a relationship between the severity of airway obstruction and the extent of VVCD; therefore, similar conclusions to the human literature cannot be drawn for this population of dogs. The discrepancies between the present findings and the human literature may relate to interspecies anatomical differences. Body position is known to influence intrathoracic and intra‐abdominal pressures [27]; therefore, the quadrupedal posture of dogs, compared with the bipedal posture of humans, is likely to produce a different distribution of these pressures and, consequently, different physiological responses when they are increased. Additionally, none of the chronic comorbidities investigated in humans resulted in airway resistance; therefore, their influence on intrathoracic and intra‐abdominal pressures could be different from the physiologic mechanisms seen in dogs with BOAS. Thus, the potential role of chronically increased negative airway pressures in the pathogenesis of vascular canal dilations in dogs remains speculative and warrants further investigation.

The difference in median vertebrae affected between breeds was statistically significant, with French Bulldogs showing the lowest prevalence of VVCD amongst the three brachycephalic breeds and the English Bulldogs the highest. French Bulldogs also had fewer vertebrae affected with VVCD type “B” compared with Pugs and type “C” compared with English Bulldogs. These results are in agreement with previously reported prevalence of VVCD in English and French Bulldogs, as well as with previous studies on congenital vertebral anomalies in brachycephalic dogs [10, 11, 25]. Previous reports of congenital vertebral anomalies indicated Pugs are the least affected breed when compared with French and English Bulldogs [10, 11, 28]. This was not verified in the present study, where Pugs had fewer vertebrae affected by VVCD than English Bulldogs but more than French Bulldogs. As with other congenital vertebral anomalies in brachycephalic dogs, the differences between breeds likely reflect variations in skeletal morphology, genetic, and heritability factors [8, 29, 30].

In humans, males and patients over 60 years of age were recently shown to have lower vertebral vascular canal depth when compared to females and younger individuals [31]. This was not the case in the present study, where the patient's age and sex were found to have no significant relationship with the number or type of VVCD. The lack of association between age and VVCD could reflect the young age of patients included in the study. An initial statistical association was observed between body weight and both the number and type of VVCDs. This relationship was considered likely to reflect the influence of breed, as English Bulldogs had the highest mean body weight, the greatest number of affected vertebrae, and the highest proportion of VVCD yypes “B” and “C”. This interpretation was supported by the multivariable analysis, which showed that, after controlling for body weight, breed remained the only significant factor.

Although CT following surgical intervention for BOAS was only performed in a very small number of cases, the lack of change in the VVCD type or number, associated with a reduction in BOAS grading, suggests that these anomalies are likely to develop early in life and remain static despite treatment. Another important consideration is that the present study is limited to assessment of the osseous structures. If the observed malformation results from chronically elevated intracanal pressure—whether due to mechanisms such as pressure‐induced bone resorption or gradual expansion—the bony architecture is unlikely to return to its original morphology. However, this does not necessarily imply that the intracanal pressure has remained unchanged. To evaluate potential alterations in soft tissue structures, such as vascular dimensions within the vascular canal, further imaging—preferably magnetic resonance imaging rather than CT—may be required. The use of contrast‐enhanced magnetic resonance angiography has been considered a feasible technique to evaluate the canine lumbar spinal cord vascular supply [12]. A similar technique could be considered to visualize the thoracic VVS in normal dogs and dogs with VVCD.

It has previously been suggested that excessive dilation of the basivertebral veins after their formation may result in abnormalities in the conformation of the vertebral vascular canal [12]. The present study did not identify a significant relationship between BOAS severity and the type of VVCD, and thus the hypothesis that chronic venous congestion due to airway obstruction and consequent changes in thoracic and abdominal pressure may exacerbate this anomaly could not be supported. The exact etiology of VVCD remains speculative.

A key limitation of this study is the potential selection bias inherent in using a referral population, likely skewing the sample toward dogs with more severe BOAS (grades II and III). Although no correlation was identified between the presence or type of VVCD and BOAS grading, intrathoracic and intra‐abdominal pressures were not measured in this study; therefore, the absence of a relationship with pressure changes can only be considered speculative. Intrathoracic or intra‐abdominal pressures are not routinely measured in dogs, particularly due to their invasiveness; however, this can be considered in future prospective studies and may provide further information regarding the etiology of VVCD [32, 33, 34]. The selection of patients for the control group is also a limitation of the study, as animals were included regardless of their primary complaint. It is possible that dogs with thoracic disease may have been included, which could plausibly have altered their intra‐abdominal or intrathoracic pressures. Another limitation of this study is the potential for observer bias, as all measurements were obtained by a single author under the supervision of an ECVDI‐certified radiologist. To address this limitation, interobserver agreement analysis was performed. Agreement was moderate for the number of VVCDs identified and fair for both the vertebrae affected and the VVCD type classification. These findings highlight potential limitations in measurement validity and reproducibility. Further studies evaluating intra‐ and interobserver reliability are warranted to better validate the measurement methodology.

In conclusion, the current study reaffirms the high prevalence of VVCD in brachycephalic breeds; however, it failed to identify a significant relationship between VVCD number and type and BOAS severity. Further research into the pathophysiological mechanisms linking airway obstruction and vertebral anomalies is essential to inform interventions and improve the welfare of brachycephalic dogs.

## Author Contributions

**Thomas Maddox**: formal analysis, writing – review and editing. **Margarida de Almeida Coelho**: writing – original draft, writing – review and editing, data curation, formal analysis, methodology. **Marie‐Aude Genain**: conceptualization, methodology, writing – review and editing, formal analysis. **Jane F. Ladlow**: writing – review and editing, supervision.

## Disclosure

Preliminary findings from this study were presented at the 2024 European Association of Veterinary Diagnostic Imaging‐British and Irish Division Spring Meeting. A reporting checklist was not used.

## Conflicts of Interest

The authors declare no conflicts of interest.

## Data Availability

The data that support the findings of this study are available from the corresponding author upon request.
